# Acute Myocardial Infarction following Naltrexone Consumption; a Case Report

**Published:** 2017-01-14

**Authors:** Bita Dadpour, Arash Gholoobi, Shahrad Tajoddini, Amir Habibi

**Affiliations:** 1 Addiction Research Centre, Imam Reza Hospital, Mashhad University of Medical Sciences, Mashhad, Iran.; 2 Cardiac Anesthesia Research Centre, Imam Reza Hospital, Mashhad University of Medical Sciences, Mashhad, Iran.; 3 Atherosclerosis Prevention Research Center, Imam Reza Hospital, Mashhad University of Medical Sciences, Mashhad, Iran.; 4 Neuroscience Research Center, Institute of Neuropharmacology, Kerman University of Medical Sciences, Kerman, Iran.

**Keywords:** Naltrexone, myocardial infarction, substance withdrawal syndrome, narcotic antagonists

## Abstract

Cardiovascular effects of opioid withdrawal have long been studied. It was reported that patients with underlying ischemic heart disease and atherosclerotic vessels may be complicated by a sudden physical and emotional stress due to withdrawal syndrome. But some other believes sudden increase in catecholamine level as a sympathetic overflow might effect on heart with and without underlying ischemia. In the current study, a patient on methadone maintenance therapy (MMT) who experienced myocardial infarction (MI) after taking naltrexone was described.

## Introduction

Cardiovascular effects of opioid withdrawal have long been studied. Opioid withdrawal induces agitation, muscular pain, vomiting, diaphoresis, rhinorrhea, mydriasis as well as tachycardia and hypertension, which could be related to a transient increase in catecholamines. Older patients with underlying cardiac ischemia could be at greater risk for cardiac events following abrupt withdrawal(1, 2); although this issue has been challenged(3). It was reported that patients with underlying ischemic heart disease and atherosclerotic vessels may be complicated by a sudden physical and emotional stress due to withdrawal syndrome. But some other believes sudden increase in catecholamine level as a sympathetic overflow might effect on heart with and without underlying ischemia.In the current study, a patient on Methadone Maintenance Therapy (MMT) who experienced myocardial infarction (MI) after taking naltrexone was described.

## Case Reports

A 64-year-old man was admitted in emergencytoxicology ward with altered mental status and agitation. His symptoms were started abruptly following consumption of naltrexone and were progressed to respiratory distress and tachypnea. On medical history, he was diabetic and hypertensive. He was also on regular consumption of methadone (40 milligram daily) for recent 4 years following 20 years opium addiction. 

**Figure 1:**
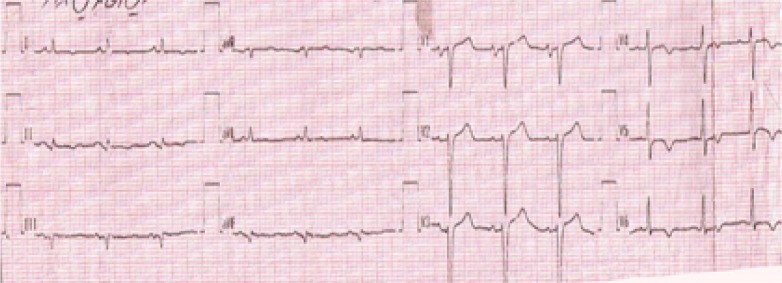
Electrocardiogram on arrival

**Figure 2:**
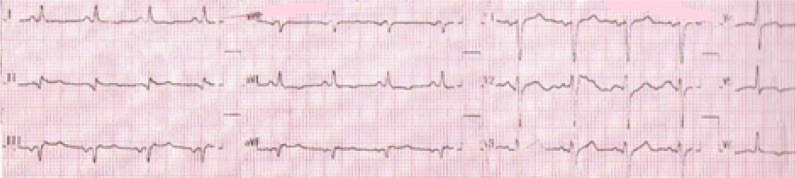
Electrocardiogram 12 hours after admission

He had blood pressure 170/110 mmHg,heart rate: 90/minute, respiratory rate 24/minute, tympanic temperature 36.8°C, and serum blood sugar 504 mg/dL on arrival. Due to decreased level of consciousness and to maintain the airway, he was intubated and underwent mechanical ventilation. Electrocardiography (ECG) on admission revealed old silent inferolateral myocardial infarction (MI) that showed in figure 1.Intravenous nitroglycerin and regular insulin were administered for controlling high blood pressure and hyperglycemia and he was admitted in intensive care unit (ICU). After a while, his blood pressure reached to 200/150mmHg and dynamic changes were occurred on the ECG as revealed in figure 2, that illustrate an acute inferior myocardial infarction associated with positive troponin-I level. Bedside echocardiography revealed global hypokinesis along with akinesis in apex and inferior wall and severe hypokinesis in inferior septal and anteroseptal wall. Left ventricular ejection fraction reported as 30-35%. Systolic pulmonary arterial pressure reported 30 mmHg and no pericardial effusion was detected. Unfortunately, percutaneous coronary intervention was not accessible, so medical treatment for acute coronary syndrome was started based on recommendations of cardiologist consult.He was referred to cardiology ward in day 15 for further work up, where coronary angiography was performed and revealed three vessel diseases. He underwent coronary artery bypass grafting (CABG) on day 16 and finally was discharged after 27days with recommendation to related cardiologic follow up in outpatient clinic. 

## Discussion

Potential relationship between opioids and coronary artery diseases has been widely studies in humans and animals(4-6). Opioid induced manifestation on coronary artery disease (CAD) is controversial and ranges from protective effects to triggering role in patients with coronary artery disease (1).It was reported that opium consumption may be positively correlated with the risk of CAD in diabetic opium addict subjects undergoing coronary angiography. This effect was dose dependent (7). Abrupt discontinuation of opioids or consumption of opioid antagonists in addict subjects lead to opioid withdrawal syndrome. This syndrome is considered to be a true physical stress and presented with agitation, severe muscular pain, vomiting, diaphoresis, rhinorrhea and mydriasis. A transient increase in catecholamines may also cause tachypnea, tachycardia and hypertension, which has been described as an overshoot phenomenon (8, 9). Altered mental status might also occur. Naltrexone, μ- and κ-receptor antagonist,with a half-life of 10 hours could induce withdrawal symptoms if administered in opioid dependent cases. Clinical manifestations of withdrawal syndrome usually appear within five minutes after consumption of Naltrexone. (10).

Catecholamine release could lead to myocardial stunning and impaired perfusion of coronary flow reserve (11). Reduced subendocardial perfusion, pulmonary edema and cardiac arrhythmias are being reported to be related to catecholamine release in opioid withdrawal (12, 13). Increased noradrenergic and dopaminergic activity and consequent effects on heart following opium antagonist agents administration to morphine-dependent subjects has also been described in previous animal researches (14-16). Potential mechanisms could be as follows: decrease in coronary flow reserve; microvascular dysfunction; direct effects of catecholamineson cardiac myocytes through calcium overload mediated by cyclic AMP; oxygen-derived free radicals; contraction band necrosis which is an interstitial mononuclear inflammatory response; thrombosis formation in context of atherosclerotic vessels; increased blood pressure and ventricular contractility (17-19).

This case report describes a methadone addict individual with underlying ischemic heart disease who experienced MI following naltrexone consumption. MI occurred in early phase of withdrawal syndrome. Naltrexone induced physical and emotional stress in a patient on MMT may cause acute coronary syndrome in a patient with underlying ischemic heart disease. Naltrexone should never be prescribed to an opioid addict; If happens, severe withdrawal syndrome will occur (8, 9). Opioid addict cases on MMT and their family should be educated for this serious complication.It is recommended that naltrexone should be used 10 to 14 days after the last dose of methadone or at least 7 to 10 days after opium discontinuation(20-22). 
